# Physiological impacts of ABA–JA interactions under water-limitation

**DOI:** 10.1007/s11103-016-0503-6

**Published:** 2016-06-14

**Authors:** Carlos de Ollas, Ian C. Dodd

**Affiliations:** Lancaster Environment Centre, Lancaster University, Lancaster, UK

**Keywords:** ABA, JA, Drought tolerance, Crosstalk, Signalling, Stomatal closure

## Abstract

Plant responses to drought stress depend on highly regulated signal transduction pathways with multiple interactions. This complex crosstalk can lead to a physiological outcome of drought avoidance or tolerance/resistance. ABA is the principal mediator of these responses due to the regulation of stomatal closure that determines plant growth and survival, but also other strategies of drought resistance such as osmotic adjustment. However, other hormones such as JA seem responsible for regulating a subset of plant responses to drought by regulating ABA biosynthesis and accumulation and ABA-dependent signalling, but also by ABA independent pathways. Here, we review recent reports of ABA–JA hormonal and molecular interactions within a physiological framework of drought tolerance. Understanding the physiological significance of this complex regulation offers opportunities to find strategies of drought tolerance that avoid unwanted side effects that limit growth and yield, and may allow biotechnological crop improvement.

## Introduction

At both tissue and whole plant levels, abscisic acid (ABA) is the most important hormone controlling plant water loss and hence plant water status and performance in water limited conditions. ABA is the main driver of stomatal closure which is probably the most effective mechanism to minimise dehydration. In terms of water stress sensing, stomata and roots are the most important organs that integrate environmental conditions with plant water status, with an important distinction depending on the origin (root or leaf) of the stress sensation (Kramer 1988; Passioura 1996). Both soil moisture and vapour pressure deficit (VPD) can influence plant responses to water stress, even if these environmental conditions have a similar outcome (e.g. stomatal closure). In response to drought, plants can exhibit a strategy of drought escape/avoidance or drought tolerance. Avoidance implies the maintenance of tissue water potential even in conditions of low soil moisture whereas tolerance indicates the ability to withstand water deficits that lower tissue water potential. Mechanisms of drought tolerance imply the biosynthesis of functional proteins in response to drought like molecular chaperones, hydrophilic proteins [dehydrins and late embryogenesis abundant (LEA) proteins] and enzymes involved in the synthesis of osmoprotectants (proline and sugars). Roots “sense” soil drying thereby upregulating ABA synthesis, some of which may be transported in the xylem to the shoots (Wilkinson and Davies 2002; Puértolas et al. 2013). Leaves must integrate multiple environmental signals including these chemical/hydraulic signals transported via the xylem sap, as well as light, CO_2_ concentration, VPD and temperature.

At the molecular level, there are thousands of ABA dependent genes controlling growth, senescence, secondary metabolism, protein biosynthesis and osmotic adjustment to coordinate plant behaviour under stress conditions (Yoshida et al. 2014). Classically, these studies of drought related gene expression have been divided into ABA-dependent and ABA-independent branches of water stress signalling (extensively reviewed by Yamaguchi-Shinozaki and Shinozaki 2006; Roychoudhury et al. 2013; Yoshida et al. 2014). A mosaic of transcription factors (TFs) and genes control several pathways and modify expression of the corresponding ABA-responsive or water stress-responsive genes. Nevertheless a more subtle network of water stress signalling has emerged in the last decade, with other hormones like jasmonic acid (JA), ethylene, auxin, cytokinin and brassinosteroids playing diverse roles to fine tune ABA biosynthesis and hence water stress signalling/plant physiological responses. This can be defined as hormonal and molecular crosstalk.

Most of the molecular knowledge of plant responses to water deficit comes from laboratory experiments where plants experience dehydration due to water withdrawal (Harb et al. 2010; Cheng et al. 2013) or osmotic treatments (Weng et al. 2011; Liu et al. 2012). While these experiments unveiled genetic networks of stress signalling, plant genotypes can be wrongly classified as stress tolerant if they survive by avoiding dehydration (by limiting water losses). Measuring plant water status (tissue water content/water and osmotic potential/turgor) is necessary to determine if plants show drought tolerance (improved performance at the same water status). Understanding both hormonal crosstalk and physiological responses are necessary if agronomists are to exploit drought avoidance/tolerance mechanisms in farmers’ fields. Lately, JA has gained much attention as it participates in abiotic stress responses [recently reviewed by Kazan (2015)] in addition to its involvement in biotic stress (Wasternack 2014). Jasmonic acid is an attractive topic of research due to its participation in multiple stress signalling responses. For example, in biotic stress it is well known that JA plays an antagonistic role to salicylic acid but works synergistically with Ethylene (ET) (Hirayama and Shinozaki 2010). In contrast, the relation of JA with other hormones (mainly ABA and ET) in response to abiotic stress is still being studied. The fact that JA presents such a multifaceted behaviour and is implicated in a broad range of common stress elicitors are powerful reasons to draw our attention, especially if manipulating these interactions offers the possibility to improve crop tolerance to both biotic and abiotic conditions. By focusing on ABA-JA interactions, this article aims to summarize the participation of JA in plant physiological responses and its molecular regulation to drought leading to tolerance/adaptation. We briefly summarize the molecular interactions of ABA and JA signalling leading to meaningful physiological responses to drought.

## Tissue ABA/JA accumulation

ABA accumulates in shoots (Zhang et al. 2012) and roots (Puértolas et al. 2013) even under optimal conditions, but this accumulation is stimulated by any decrease in cellular turgor. The initial mechanism linking the stress sensation and ABA biosynthesis is still unknown, but ABA biosynthesis under stress conditions is directly correlated with plant/tissue water status. Although ABA has many biosynthetic steps [as described in Nambara and Marion-Poll (2005)], NCED seems to be the key biosynthetic enzyme in the pathway (Thompson et al. 2007) because its transcription is well correlated with plant water status and mutants that overexpress NCED, and lines transformed with multiple copies of NCED accumulate more ABA (Thompson et al. 2000). Nevertheless, local ABA concentration is a balance between biosynthesis, catabolism and transport between cells and tissues (e.g. root-to-shoot) (Nambara and Marion-Poll 2005; Jiang and Hartung 2008; Munemasa et al. 2015).

Jasmonic acid is synthesized via the octadecanoid pathway under multiple (both biotic and abiotic) stress conditions, and is associated with resistance to biotic stress and wounding in stress signalling. Of the JA precursors, 12-oxophytodienic acid (OPDA) is one of the most important due to its possible independent roles in signalling downstream of JA. The Isoleucine conjugate form of JA, jasmonic acid Isoleucine (JA-Ile) has been characterized as the key signalling compound in JA-dependent responses due to its interaction with the receptor Coronatine-Insensitive 1 (COI1). Several recent publications have demonstrated JA’s involvement in water stress responses (Brossa et al. 2011; De Ollas et al. 2015). JA and ABA seem to share common targets and crosstalk between both signalling pathways is extensive. JA application (5–15 µM in hydroponic solution) enhanced foliar ABA concentration 4–7-fold (Bandurska et al. 2003) and JA deficiency (*jar*1) can diminish ABA accumulation (De Ollas et al. 2013). It is important to point out that ABA concentration usually increases as tissue water status declines (Puértolas et al. 2013), while JA concentration fluctuates and seems to accumulate during early stages of stress. While JA accumulation in response to dehydration has been suggested (Arbona et al. 2010), its involvement in molecular and physiological responses, either in concert with ABA or acting independently, is still unclear. Importantly, after transient JA accumulation, JA-dependent signalling may remain active (Wasternack 2014).

## Molecular responses to ABA/JA

**Fig. 1:**
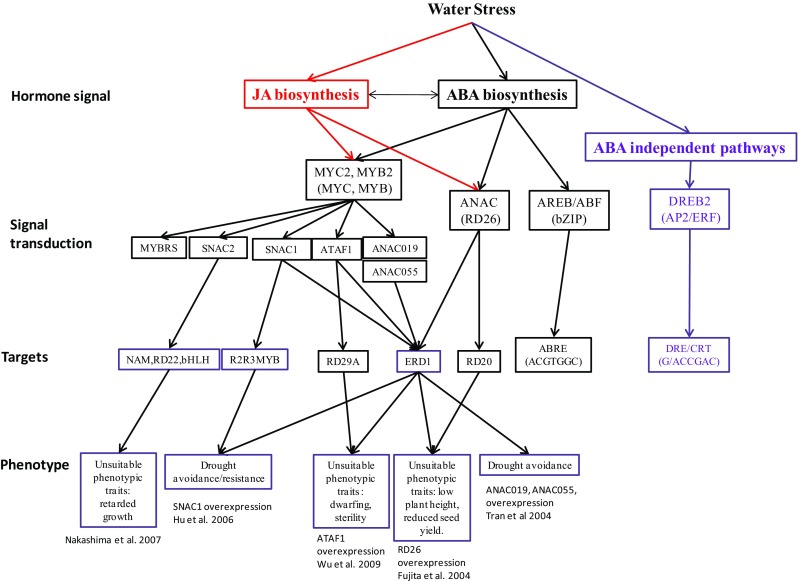
Summary of signalling pathways related to JA–ABA interaction in response to drought. Briefly, water deficit can enhance both ABA and JA accumulation. Water stress also activates ABA independent pathways via DREB2 transcription factors targeting genes with DRE sequences in their promoters. The ABA dependent pathway uses AREB/ABF, ANAC and MYC transcription factors. AREB seems to be ABA-specific targeting ABRE sequences, ANAC and MYC seem to be activated by JA and ABA. Overexpression of each of these transcription factors activates a subset of genes giving a specific phenotype of “drought tolerance” indicated at the base of the figure

ABA-dependent signalling has several branches controlled by ABA-responsive elements (ABREs), NAC and MYC/MYB TFs (Fig. 1—Signal transduction). The ABRE-branch of gene expression requires multiple ABREs, or the combination of an ABRE with a coupling element (Zhang et al. 2005). Usually a single ABRE is insufficient for the ABA dependent initiation of transcription; successful initiation requires another ABRE element or another type of coupling element like CE1 (coupling element 1), CE3, motif III, or a drought-responsive element (DRE)/CRT (Umezawa et al. 2010). Among the group AbZIP subfamily, AREB1/ABF2, AREB2/ABF4 and ABF3 are master TFs that cooperatively regulate ABRE-dependent gene expression in ABA signalling under stress conditions. In addition to AREB/ABF, there are other TFs involved in ABA-mediated gene expression in response to abiotic stress. The AP2/ERF proteins play a central role in stress signalling (Liu et al. 2000), while the subfamily of DRE/CRT binding proteins (DREB1/CBFs) can bind to the DRE cis-acting element. DREB1D/CBF4 and the cold-responsive DREB1A-C/CBF1-3 are also induced by exogenous ABA. On the other hand, DREB2A and DREB2B are strongly induced by osmotic stress but only slightly by ABA (Umezawa et al. 2010). This requirement for multiple heterogeneous or homogeneous elements of regulation offers the possibility of controlling transcription by tuning the ABA concentration in a tissue (two ABRES) or initiating transcription by the action of ABA and another signalling input (ABRE and a coupling element). This system is more plastic and better able to adapt to specific environmental conditions.

On the other hand, JA dependent signalling is initiated by the interaction of JA-Ile with the receptor COI1 which releases a series of molecular responses that involve interactions with many signalling components including the JAZ proteins and MYC2 TF’s (reviewed by Wasternack 2014). ABA and JA signalling pathways can interact at several points, suggesting a role for JA in the response to water stress. MYC2 and MYB2 TF’s are involved in ABA-mediated gene expression in Arabidopsis (Kazan and Manners 2013). Transgenic plants overexpressing MYB2 and MYC2 show enhanced sensitivity to ABA and have lower electrolyte leakage following cessation of watering (Abe et al. 1997, 2003), likely because enhanced stomatal closure limits dehydration. The induction of MYC2 by ABA seems to rely on the JA receptor COI1 (Lorenzo et al. 2004). In *Oryza sativa*, the MYC-family TF OsbHLH148 interacted with OsJAZs in response to drought (Seo et al. 2011). A model for the OsbHLH148-related jasmonate signalling pathway in drought stress was proposed such that ABA and JA act synergistically in response to stress with JA acting upstream of ABA.

Overexpressing OsbHLH148 plants reportedly had a drought tolerant phenotype associated with the expression of OsDREB1. Transformed rice plants had a higher survival rate (77–92 %) than WT plants (9 %) when water was withheld, and detached air-dried leaves had a higher photosystem II efficiency (Fv/Fm) than non-transformed plants. This suggests greater stomatal closure to maintain leaf water status and protect the photosystems. It is uncertain whether these specific mechanisms will be relevant to maintaining yields in dryland rice production. In contrast, another publication dealing with MYC2-related signalling reported that after ceasing watering, then maintaining a moderate drought by daily irrigation (leaf relative water content (RWC) averaged 65 %), both *coi*-1 (insensitive to JA) and *jin*-1 (insensitive to the MYC2 dependent branch of JA signalling) mutants showed no biomass reduction while this treatment approximately halved biomass of WT and *jar*1 plants (Harb et al. 2010). This response was unrelated to the biomass (and presumably transpiration rate) of *coi*-1 plants, which grew similarly to WT plants under well-watered conditions. Thus perturbing JA signalling may enhance biomass accumulation, but further work is required to evaluate the generality of this response, since any trait may confer drought tolerance under specific drought conditions (Tardieu 2012).

The Ethylene (ET) transcription factor family are interesting targets for both JA-ABA interactions and ET. ERF5 and ERF6 are two redundant TF that are involved in leaf growth inhibition under mild drought stress (Dubois et al. 2013), with the expression of these genes being JA/ET dependent (Moffat et al. 2012). In contrast, ERF11 (responsive to ABA and JA) acts antagonistically to ERF6, with ABA inactivating ERF6 under water deficit (Sewelam et al. 2013). Another member of ERF family, ERF1, can be activated by ET, JA or by the synergistic combination of both hormones (Lorenzo et al. 2003) and seems a point of convergence of both hormonal pathways. Although ERF1 overexpression reportedly enhanced drought tolerance, no details of the drought selection were explained (Weiste et al. 2007). When 35S:ERF1 plants displayed resistance to various stresses (high salinity, drought and mannitol), ERF1 expression increased rapidly (within 1–2 h of stress imposition) and transiently (Cheng et al. 2013), similar to the pattern of JA accumulation in response to dehydration (De Ollas et al. 2013). Interestingly, foliar spraying with 50 µM ABA decreased ERF1 expression, thereby overriding JA and ET induction. Since the drought resistance phenotype was based on plant survival rate following water withdrawal and recovery (90 % for overexpressing lines versus 33 % for WT), overexpressing lines avoided drought by reduced transpiration rate due to enhanced stomatal closure. Moreover, stomata of 35S:ERF1 plants were more closed even in well-watered conditions, probably implying a lower growth rate in optimal conditions. Also, these plants seem to accumulate more proline and ABA in well-watered conditions, an undesirable trait in any crop according to a comprehensive analysis of various conceptual ABA ideotypes (Blum 2015). ERF has 122 members in Arabidopsis, and the complexity of the interaction of this network with ABA-JA-ET and other players in abiotic conditions makes it difficult to create useful models (synergism *versus* antagonism of ABA-JA in water stress and interaction with ET) requiring caution in assessing phenotypes.

Several NAC TF’s are upregulated by ABA, salinity and dehydration and it is possible that ANAC055 and RD26/ANAC072 proteins bind to the CATGTG motif and regulate stress inducible genes associated with drought tolerance. ANAC019 and ANAC055 are active in E3 ligase modulation of ABA signalling, playing dual roles in regulation of ABA and JA responses (Fujita et al. 2011). Since both of these TF’s are involved in both JA and ABA signalling (Fig. 1), ABA- and JA-insensitive mutants are necessary to discriminate the pathways involved in drought tolerance. ABA-induced expression of ANAC019 and ANAC055 is impaired in *coi1*-*1* and *jai1/jin1* (Jiang et al. 2009), reinforcing the participation of those TFs in crosstalk between JA and ABA pathways. Moreover, an involvement of ANAC019 and ANAC055 in drought stress regulation was reported because they bind specifically to the promoter region of ERD1, a dehydration responsive element. More recently, constitutive overexpression of a *Miscanthus lutarioriparius* NAC gene (MINAC5) enhanced drought and cold tolerance in Arabidopsis by inducing ABA hypersensitivity (Yang et al. 2015), as in overexpression of other TF’s such as OsbHLH148. If these TF’s are able to improve mechanisms allowing higher photosynthesis even when stomata are more closed (higher water use efficiency) due to protective mechanisms under low soil moisture, they may improve drought tolerance.

Several examples of genuine drought tolerance associated with the NAC family of TF exist, and the effects of NAC overexpression under stress conditions has been reviewed (Puranik et al. 2012). SNAC-1 (STRESS RESPONSIVE NAC 1) is upregulated upon treatment with both ABA and JA (Nakashima et al. 2012). Overexpressing SNAC-1 in rice plants delayed leaf rolling and reduced water loss (drought avoidance) in field-grown plants at anthesis (Hu et al. 2006). Interestingly, despite having up to 20 % lower stomatal conductance than WT plants, photosynthetic rate was similar to WT plants, and these overexpressing plants re-established turgor pressure at a lower RWC upon re-watering, suggesting enhanced drought tolerance was conferred by improved osmotic adjustment. On the other hand, constitutive overexpression of OsNAC6 (SNAC-2) in rice retarded growth and decreased productivity, although survival rate after desiccation was higher (Nakashima et al. 2007). OsNAC6/SNAC-2 is induced by cold, salt drought, ABA and JA (Ohnishi et al. 2005). However, root specific (RCc3 promoter) overexpression of the NAC family *Os*NAC10 in *Oryza sativa* significantly enhanced drought tolerance at the reproductive stage, increasing grain yield by 25–42 % and by 5–14 % over controls in the field (with more filled grains and spikelets) under drought (rain was excluded from 10 days before heading to 20 days after heading) and normal conditions respectively (Jeong et al. 2010). This may be related to a more extensive root system, thereby maintaining water uptake. In contrast, constitutive (GOS2) overexpression of *Os*NAC10 throughout the plant (including the floral organs) greatly decreased grain filling rate under both normal and drought conditions. Since improved root development is a common strategy for drought avoidance; using root specific promoters (Ghanem et al. 2011) may represent a practical strategy to harness beneficial NAC-dependent traits without some of the deleterious side-effects resulting from constitutive overexpression.

Due to space limitations we have not addressed the involvement of WRKY TF family involvement in this topic, partly because of the complexity of the family and the multiple interactions. Nevertheless recent work illustrates the involvement of WRKY TF family in ABA-JA response to drought stress (Rabara et al. 2015).

## Physiological responses to ABA/JA

**Fig. 2:**
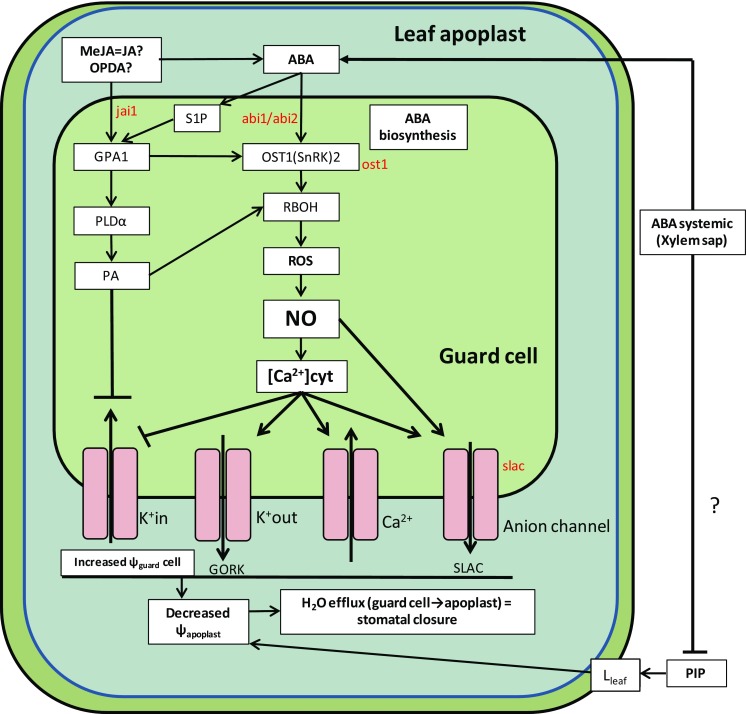
Stomatal closure signalling pathways. ABA produced in the guard cells or imported to the apoplast from other tissues unleashes the production of NO and ROS, the subsequent release of Ca^2+^ controls the activity of several ion channels causing decreased osmotic pressure in the cell, leading to H_2_O efflux and stomatal closure. This pathway can be activated by MeJA. Question marks highlight the lack of information on the individual activity of JA, MeJA and OPDA activating this pathway or modifying ABA biosynthesis. An alternative hydraulic pathway activated by ABA (Pantin et al. 2013) is described where ABA deactivates PIP aquaporins in the bundle sheath decreasing leaf hydraulic conductivity (L_leaf_), which will decrease ψ_leaf_ and g_s_. Question marks reflect steps that are theorized but not sustained by experimental data. Mutants in red designate an interruption in the pathway that has been experimentally verified

Plants cope with water stress by closing their stomata to avoid water loss. Increased ABA concentrations induce stomatal closure via intracellular signalling in the guard cells. Cytosolic production of nitric oxide (NO) and reactive oxygen species (ROS) (Fig. 2), due to the interaction of ABA with PP2C (ABA receptor) and SnRK2 (ABA dependent phosphorylase), causes fluctuations in symplastic Ca^2+^ concentrations (induced by the activity of several ion transporters). This causes ion efflux thereby lowering cellular turgor due to water efflux out of the guard cells and hence causing stomatal closure. This signal transduction pathway, along with involvement of JA and other hormones was recently reviewed (Daszkowska-Golec and Szarejko 2013). In addition to the classical direct involvement of ABA-mediated stomatal closure via ABA signalling in the guard cells, it can also indirectly induce stomatal closure by decreasing leaf hydraulic conductance (Pantin et al. 2013). While the ABA insensitive mutants *abi1*-1 and *abi2*-1 displayed stomatal closure when detached leaves of Arabidopsis were fed 50 µM ABA via the transpiration stream, the *slac*1-1 and *ost*2-2 mutants (Fig. 2) were insensitive to ABA (Pantin et al. 2013). To explain this disparity and the fact that plants defective in ABA signalling closed their stomata under high VPD, ABA inactivation of bundle sheath aquaporins (PIPs) was postulated based on (Shatil-Cohen et al. 2011). Briefly, an ABA dependent signalling pathway decreases PIP aquaporin activity thus reducing leaf hydraulic conductance and decreasing leaf apoplast water potential (Fig. 2), although whether ABA inhibits or stimulates hydraulic conductance is concentration-dependent (Dodd 2013). This work suggests an unexplained ABA signalling that is independent of ABI1-2, ABI2, SLAC and OST in decreasing leaf conductance, and that ABA may modify other hormonal pathways thereby affecting PIP activity. Due to the extensive hormonal crosstalk that can influence stomatal closure, excluding the influence of other hormones would further establish this interesting model of chemical/hydraulic regulation.

It is well established that ABA plays a central role in regulating stomatal closure (Daszkowska-Golec and Szarejko 2013; Dodd 2013) yet other phytohormones can also have direct effects and/or mediate the influence of ABA. JA’s involvement in stomatal closure is not completely understood, even though many experiments have exposed isolated epidermis to various agents (Table 1). Exogenous application of either ABA or MeJA to detached leaves elicited stomatal closure (Suhita et al. 2004). In contrast, it was suggested that MeJA needs a pool of ABA to be effective in closing stomata, since MeJA treatment was ineffective in closing stomata in isolated epidermis of ABA deficient mutants (*aba*) and plants treated with the ABA biosynthesis inhibitor fluridone (Hossain et al. 2011). Thus MeJA may exercise its action by increasing ABA biosynthesis, as previously suggested (Kim et al. 2009) in MeJA overaccumulating rice. Alternatively, MeJA treatment did not elicit stomatal closure in ABA deficient mutants but ABA treatment did; suggesting that ABA acts downstream of *coi1*, while in *abi*1-2 neither ABA nor MeJA elicited stomatal closure. The authors suggested a model of independent MeJA and ABA signalling converging upstream of the second messengers (ROS and NO) induction (Munemasa et al. 2011). A schematic of these interactions is summarised in Fig. 2.

**Table 1:** Summary of stomatal closure following ABA, MeJA and OPDA exogenous treatments to epidermal peels of Col-0 or Landsberg erecta

Hormonal treatment	Concentration (uM)	Stomatal closure (%)	Genotype	Source
ABA	1	50	Col-0	Savchenko et al. (2014)
ABA	10	80	Col-0	Montillet et al. (2013)
ABA	1	30	Col-0	Munemasa et al. (2007)
ABA	10	25	Col-0	Hossain et al. (2011)
ABA and MeJA	1	50	Col-0	Montillet et al. (2013)
ABA or MeJA	10	70	Ler	Suhita et al. (2004)
MeJA	50	50	Col-0	Savchenko et al. (2014)
MeJA	1	n.s.	Col-0	Montillet et al. (2013)
MeJA	1	25	Col-0	Munemasa et al. (2007)
MeJA	10	25	Col-0	Hossain et al. (2011)
12-OPDA	10	50	Col-0	Savchenko et al. (2014)

*ns* not significant

However, there are also some discrepancies in the direct effect of exogenous treatment of MeJA on stomatal closure (Table 1). In Arabidopsis (Col-0), MeJA only reduces stomatal aperture at concentrations exceeding 200 µM (Savchenko et al. 2014) whereas almost complete stomatal closure occurred in epidermal peels of Arabidopsis (Ler and Col-0) treated with either MeJA or ABA at much lower concentrations (10–20 µM) (Suhita et al. 2004). In contrast to these results and also in epidermal peels of Arabidopsis, concentrations up to 100 µM MeJA had no effect on stomatal aperture but lower concentrations of OPDA (precursor of JA) decreased stomatal aperture (Montillet et al. 2013). According to Savchenko’s model, OPDA promotes stomatal closure independently from JA and in cooperation with ABA. Nevertheless, treatment with 1–10 µM of ABA or MeJA (Suhita et al. 2004) was much less effective in closing stomata of the *jar*1-1 mutant (defective in conjugating isoleucine to JA), suggesting that MeJA treatment is JA-Ile dependent and hence COI-1 dependent. These experiments expose the variability of the stomatal responses to exogenous hormonal treatment and the necessity of assessing apoplastic hormone concentration adjacent to the guard cells to better compare epidermal bioassays with *in planta* experiments. This is important to distinguish hormonal crosstalk (JA influencing ABA biosynthesis in this particular example) from molecular interactions in a signalling pathway leading to a physiological outcome (degree of stomatal closure).

Although water uptake is controlled by stomatal opening, changes in root hydraulic conductivity (L_pr_) can also influence plant water status to mediate transpiration. While it is important not to underestimate the importance of root morphology and anatomy in mediating L_pr_ (Vadez 2014), regulation of cell-to-cell water transport might also play an important role in maintaining water status under moderate water stress. Nevertheless, soil water conductivity is likely more limiting under severe water deficits (Draye et al. 2010). Several publications highlighted the involvement of exogenous ABA application (100 µM) (Aroca et al. 2008; Kudoyarova et al. 2011) and transgenic plants with enhanced root ABA concentrations (Thompson et al. 2007) in enhancing L_pr_. Nevertheless, applying higher ABA concentrations to the root system can decrease L_pr_ (reviewed in Dodd 2013). Hormonal regulation of L_pr_ is an attractive topic for further research, but since plant water status seems primarily regulated by stomatal responses, it is uncertain whether changing L_pr_ can improve plant water status thereby promoting drought tolerance.

Supplying MeJA (10–100 µM) in nutrient solution can also increase L_pr_ of WT tomato plants, while plants deficient in JA (*def*-*1*) had a lower L_pr_ (Sánchez-Romera et al. 2014). Exogenous application of 100 µM MeJA rescued this phenotype in JA deficient mutants. Although the short term response (1 h) was partially dependent on ABA (treatment with the ABA biosynthesis inhibitor fluridone partially abolished the MeJA effect), the long term effect was independent of ABA. MeJA treatment modified the expression and activity of PIP aquaporins, with the expression of some isoforms downregulated and others upregulated. Such tissue specific regulation of aquaporins by hormones under stress conditions can link hormonal dependent signalling with hydraulic regulation.

## Conclusions and future scope

While jasmonates seem involved in drought stress responses, a more comprehensive assessment of environmental and physiological responses (soil and plant water status, stomatal closure and photosynthesis) are necessary to understand the molecular mechanisms that can potentially improve crop yields in water-limited environments. Assessing ABA and JA interactions in drought stress responses needs to consider the three levels of interactions summarised in this short review (biosynthetic, molecular and physiological). Accurate hormonal quantification of ABA and JA, JA-Ile and ideally OPDA in relevant tissues is necessary (Dave et al. 2011) to assess the real players in most physiological responses, since both hormonal pathways can regulate the other, and to identify the bioactive molecule(s) in these interactions (OPDA *versus* JA and JA-Ile). We also need to assess the direct influence of jasmonates in responses to drought as distinct from the indirect influence through ABA biosynthesis and signalling. It is important to unveil these interactions because they offer an opportunity to exploit the natural complexity of drought signalling. This may allow us to enhance crop performance by inducing drought tolerance and resistance mechanisms that allow better yields in sub optimal conditions, thereby avoiding conservative strategies that sensitively close the stomata to maintain tissue water status, but limit photosynthesis thereby incurring yield penalties.
